# Advancements in Sensor Technology for Monitoring and Management of Chronic Coronary Syndrome

**DOI:** 10.3390/s25154585

**Published:** 2025-07-24

**Authors:** Riccardo Cricco, Andrea Segreti, Aurora Ferro, Stefano Beato, Gaetano Castaldo, Martina Ciancio, Filippo Maria Sacco, Giorgio Pennazza, Gian Paolo Ussia, Francesco Grigioni

**Affiliations:** 1 Department of Cardiovascular Sciences, Fondazione Policlinico Universitario Campus Bio-Medico, Via Alvaro del Portillo 200, 00128 Rome, Italy; 2 Unit of Electronics for Sensor Systems, Campus Bio-Medico University, 00128 Rome, Italy

**Keywords:** sensor, chronic coronary syndrome, technology, device, cardiovascular diseases, coronary artery disease

## Abstract

Chronic Coronary Syndrome (CCS) significantly impacts quality of life and increases the risk of adverse cardiovascular events, remaining the leading cause of mortality worldwide. The use of sensor technology in medicine is emerging as a promising approach to enhance the management and monitoring of patients across a wide range of diseases. Recent advancements in engineering and nanotechnology have enabled the development of ultra-small devices capable of collecting data on critical physiological parameters. Several sensors integrated in wearable and implantable devices, instruments for exhaled gas analysis, smart stents and tools capable of real time biochemical analysis have been developed, and some of them have demonstrated to be effective in CCS management. Their application in CCS could provide valuable insights into disease progression, ischemic events, and patient responses to therapy. Moreover, sensor technologies can support the personalization of treatment plans, enable early detection of disease exacerbations, and facilitate prompt interventions, potentially reducing the need for frequent hospital visits and unnecessary invasive diagnostic procedures such as coronary angiography. This review explores sensor integration in CCS care, highlighting technological advances, clinical potential, and implementation challenges.

## 1. Introduction

### 1.1. Overview of Chronic Coronary Syndromes

The term Chronic Coronary Syndrome (CCS) encompasses a range of clinical conditions arising from chronic diseases of the coronary arteries and/or microcirculation, leading to myocardial ischaemia. This condition is dynamic, often progressive, and can destabilize into acute events (acute coronary syndromes, ACS). Patients suffering from CCS, in fact, may experience a variable and unpredictable course, transitioning between different types of CCS and ACS presentations throughout their lifetime [1].

The prevalence of CCS and the number of deaths resulting from this condition have slowly decreased in recent years, mostly related to the primary prevention, although geographic disparities between high and low-income countries remain. However, CCS continues to represent a relevant health problem and a leading cause of death worldwide, with a prevalence of 197.2 million cases and 9.1 million deaths in 2019 [2].

Diagnosis of CCS relies on both clinical and invasive testing, including physical examination, electrocardiogram (ECG), biochemical tests, imaging such as echocardiogram, coronary computed tomography angiography (CCTA) and positron emission tomography-computed tomography (PET-CT) and invasive coronary angiography [1]. In recent years, research has focused predominantly on the prevention of coronary ischaemic events through lowering the risk of coronary artery occlusion and consequent ACS by using a guideline-directed medical therapy based on antithrombotic, lipid-lowering, anti-renin–angiotensin–aldosterone system (RAAS), anti-inflammatory, and metabolic-acting agents [3]. This approach, as previously stated, allowed for a reduction in mortality and morbidity in stable CCS [4].

In patients with CCS, long-term follow-up is fundamental to assess the risk of disease progression and to prevent the onset of a new ACS. This is usually pursued with periodical visits with the collection of ECGs and echocardiographic data. Moreover, typical CCS complications such as heart failure (HF), arrhythmias, and valvular diseases must be diagnosed and managed. Thus, a prompt recognition of new-onset symptoms or changes in ECGs and imaging suggesting disease destabilization or complication is one of the biggest challenges for the cardiologist. In this setting, new and more precise technological instruments could be helpful for the physicians.

### 1.2. Introduction to Sensors and Their Application in Healthcare

Healthcare sensors are devices capable of collecting and transmitting data related to a patient’s physiological status. These technologies are increasingly transforming the healthcare landscape by enabling continuous, real-time monitoring, which contributes to earlier diagnoses, more personalized treatment strategies, and improved management of chronic diseases.

Various medical specialties have integrated different types of sensors into their clinical routines. In diabetes care, continuous glucose monitoring (CGM) systems provide real-time glucose levels, enhancing glycemic control and reducing the risk of hypoglycemia [5]. Biosensors embedded in wearable textiles or skin patches can detect biochemical markers, such as sweat electrolytes or cortisol, supporting athletes in monitoring hydration status and stress levels [6].

Wearable sensors have also been developed for the diagnosis of obstructive sleep apnoea syndrome (OSAS), a highly prevalent and increasingly recognized disorder associated with elevated cardiovascular risk [7]. Similarly, real-time monitoring devices have been used to track physiological signals in patients with epilepsy, offering valuable data on seizure burden and treatment efficacy [8].

In patients with chronic conditions such as chronic obstructive pulmonary disease (COPD), wearable and implantable sensors can facilitate remote self-monitoring, thereby reducing the need for frequent and potentially stressful visits to healthcare facilities, events that may expose patients to exacerbation triggers [9,10,11].

In cardiology, wearable and implantable devices have long been employed, and their increasing reliability and accuracy have led major cardiology societies, both American and European, to endorse their use in recent guidelines, particularly for atrial fibrillation (AF) screening and post-ablation monitoring [12,13]. Widely available consumer devices such as the Apple Watch have shown the capability to detect AF episodes, enabling patients to document symptoms such as palpitations or syncope outside of clinical environments. This functionality enhances diagnostic yield and facilitates remote patient management [14]. Additionally, wearable blood pressure monitors and photoplethysmography (PPG) sensors offer non-invasive, user-friendly tools for early cardiovascular risk detection [15].

These examples illustrate the rapid expansion of wearable and implantable sensors across various medical disciplines. Their use supports a more personalized approach to patient management, enabling early intervention, reducing emergency department visits, and empowering patients to take an active role in managing chronic conditions. With the integration of artificial intelligence (AI), the predictive and preventive potential of these technologies is expected to grow even further.

### 1.3. Objectives of the Paper

This review aims to provide a comprehensive overview of the current use of wearable and implantable sensors in the diagnosis and management of CCS. To date, no existing review has thoroughly explored both categories of sensor technologies, encompassing continuous physiological monitoring, ischemia detection, and real-world clinical implementation, within the specific context of CCS. This review addresses that gap by focusing on the clinical applications of these sensors, evaluating their potential to improve patient outcomes through real-time physiological data collection and personalized treatment strategies. Additionally, we discuss the practical challenges and limitations associated with integrating these technologies into routine clinical practice.

## 2. Use of Sensors in Early Detection of CCS and Diagnosis

### 2.1. Rationale for Sensors’ Use in CCS Diagnosis

CCS often progresses silently, with prolonged asymptomatic phases or non-specific symptoms, which frequently delay diagnosis until the disease is at an advanced stage [16]. Such delays can lead to increased hospital admissions, greater reliance on invasive procedures, and higher healthcare expenditures.

Risk stratification plays a central role in guiding the diagnostic pathway of CCS, ensuring that diagnostic testing remains both clinically appropriate and cost-effective. This is particularly important in patients with a low pre-test probability of coronary artery disease (CAD), in whom non-invasive functional testing has been shown to be more cost-effective than invasive strategies [17].

Traditional risk stratification tools, as outlined in recent clinical guidelines, rely on static variables such as age, sex, cardiovascular risk factors, and symptom characteristics to estimate pre-test probability [18]. While effective, these models may fail to capture subtle or dynamic physiological changes that occur prior to symptom onset [19].

In the context of rapid technological advancement, sensor-based medical devices offer promising opportunities for early diagnosis and personalized care. These devices allow for continuous acquisition of physiological data in a passive, low-cost, and non-invasive manner. Given the high inter-individual variability in CCS presentation, widespread use of sensors enables a more tailored diagnostic approach by contextualizing patient symptoms within a continuous physiological framework and facilitating better prediction of disease progression. Aggregating data over time allows for dynamic risk stratification, potentially outperforming static scores based on episodic clinical encounters [20].

Patient-centered models incorporating such technologies have been associated with improved medication adherence, healthier lifestyle adoption, and a reduction in modifiable cardiovascular risk factors [19].

One of the most clinically valuable applications of these devices is the detection of silent or subclinical myocardial ischemia. Wearable ECG monitors and implantable loop recorders are capable of continuously tracking cardiac electrical activity, detecting transient ST-segment changes, and ischemic episodes that would otherwise remain unnoticed [14,21]. This is especially relevant in high-risk populations, such as individuals with diabetes, who may not exhibit typical anginal symptoms and are thus frequently underdiagnosed [22].

Several studies highlight the utility of these technologies. Shvilkin et al. [23] demonstrated that insertable cardiac monitors could detect silent ischemic episodes in asymptomatic patients with a history of CAD, leading to earlier therapeutic interventions and improved secondary prevention. Similarly, wearable ECG patches like the Zio XT Patch (iRhythm Technologies, San Francisco, CA, USA) have proven effective in detecting ST-segment changes in outpatient populations at risk for sudden cardiac death [24].

Timely recognition of myocardial ischemia significantly reduces the risk of irreversible myocardial damage and progression to HF, a condition associated with frequent hospitalizations, high costs, and substantial societal burden. Early identification and management of ischemic events through continuous sensor-based monitoring has been associated with improved clinical outcomes and a lower incidence of adverse cardiovascular events [25].

Preventing the progression to HF not only enhances patient quality of life but also alleviates pressure on healthcare systems given the high resource utilization of chronic HF management [26].

CCS is frequently complicated by cardiac arrhythmias, including AF, bradyarrhythmia, and ventricular tachyarrhythmias. High-fidelity ECG sensors can detect these abnormalities, even when they are intermittent or asymptomatic. While AF is not a direct cause of CAD, it is often associated with structural heart disease and shares common risk factors, such as hypertension, diabetes, aging, and obesity. Moreover, a high burden of premature ventricular contractions (PVCs) or other ventricular arrhythmias may reflect an ischemic substrate or previous myocardial injury.

Devices such as insertable cardiac monitors (ICMs) and advanced wearable sensors offer continuous rhythm monitoring, enabling early detection of clinically significant arrhythmias that may warrant therapeutic intervention or anticoagulation. Importantly, these technologies expand cardiovascular surveillance beyond traditional healthcare settings, making it accessible to broader populations, including individuals in underserved areas or those with limited access to in-person care [27].

By decentralizing follow-up and monitoring, sensor technologies support a more equitable and continuous healthcare model, reducing reliance on episodic clinic visits and promoting improved long-term outcomes. These systems are also highly scalable and cost-effective, which is particularly advantageous in managing chronic cardiovascular conditions that require regular monitoring. Large-scale studies have shown that remote monitoring not only enhances arrhythmia detection but also reduces hospital admissions and overall costs by enabling earlier clinical interventions [28].

Furthermore, the integration of sensor data into routine care may significantly enhance clinical research. Continuous data collection transforms each patient into a potential contributor to real-world evidence, accelerating insights into disease progression and treatment effectiveness [29].

This is exemplified by the growing use of PPG sensors embedded in consumer devices such as smartwatches. These wearables facilitate large-scale, population-level data collection. Major digital health initiatives, such as the Apple Heart Study and the Huawei Heart Study, involving hundreds of thousands of participants, have shown that wearable-based PPG can reliably detect AF and other rhythm disturbances under real-world conditions [30,31,32].

Ultimately, the integration of sensor technologies into cardiovascular care marks a paradigm shift from reactive to proactive, data-driven medicine. These tools bridge critical diagnostic gaps by enabling continuous, non-invasive, and context-aware monitoring of cardiovascular physiology. They support earlier diagnosis, individualized treatment, and more efficient use of healthcare resources.

### 2.2. Sensor Technologies in the Diagnosis of CCS

The technological landscape for diagnosing CCS is rapidly evolving, driven by advances in sensor engineering, miniaturization, and biomedical signal processing. Sensor-based diagnostic tools can be broadly classified into four main categories: wearable, implantable, biochemical, and emerging non-invasive systems. Each category offers distinct advantages, operating mechanisms, and clinical applications across various phases of the diagnostic and follow-up continuum. In Table 1, there is a schematic summary of sensor types, the technology used, and the sensors mainly studied.

#### 2.2.1. Wearable Sensors

Wearable sensors are among the most accessible and widely adopted tools in contemporary cardiovascular diagnostics. These devices, typically embedded in smartwatches, fitness trackers, chest straps, or adhesive patches, allow for non-invasive and continuous monitoring of physiological parameters. The most common modalities include electrocardiography (ECG), PPG, and phonocardiography (PCG).

##### ECG-Based Wearables

ECG sensors capture the heart’s electrical activity using skin surface electrodes. These devices are capable of detecting transient ischemic events, arrhythmias, and ST-segment deviations, key markers of myocardial ischemia and arrhythmogenic risk. While numerous models are commercially available, only a subset have received FDA clearance, confirming adherence to specific standards of safety and efficacy for medical use.

The clinical application of ECG-based wearables has been particularly focused on AF detection, due to its high prevalence, thromboembolic risk, and the feasibility of detecting irregular rhythms through consumer-grade devices. For instance, the FDA-cleared ZioPatch (iRhythm Technologies, Inc., San Francisco, California, CA, USA) provides continuous, single-lead ECG monitoring for up to 14 days. In a cohort of 26,751 patients, the device significantly improved arrhythmia detection during the initial 48 hours compared to standard Holter ECG systems [33]. The mSToPS trial further demonstrated that wearable ECG monitoring increased AF detection and the initiation of anticoagulation therapy in high-risk populations [27].

Beyond arrhythmia detection, these devices are increasingly evaluated for their capacity to detect ST-segment alterations indicative of myocardial ischemia. A study published in JAMA Cardiology validated a multichannel smartwatch ECG in detecting ST-segment elevation myocardial infarction (STEMI) and non-STEMI, showing strong agreement with standard 12-lead ECGs, with high sensitivity and specificity [34]. Ongoing trials, such as one assessing the diagnostic accuracy of a handheld six-lead ECG device in identifying inferior wall STEMI, further support the clinical relevance of wearable ECG platforms [35].

From an electronic standpoint, the use of smaller and reduced-electrode ECG wearable sensors has led to the study and proposal of numerous circuit solutions. These aim to improve performance, in terms of reliability and resolution, in order to more closely match the functionality of standard instruments and align better with clinical procedures [36,37].

##### Photoplethysmography (PPG)-Based Sensors

PPG sensors, commonly integrated into wrist-worn devices, utilize light-based technology to assess changes in blood volume and infer cardiovascular parameters such as heart rate and variability. These sensors consist of a light-emitting diode (LED) and a photodetector; the LED emits light onto the skin, and the photodetector measures the amount of light reflected or transmitted, which correlates with blood flow.

PPG enables indirect monitoring of heart rate, rhythm, and heart rate variability (HRV). Although less specific than ECG, PPG is valuable for long-term autonomic profiling. Reduced HRV has been linked to the severity of CAD and is associated with worse prognosis. Several studies have validated the prognostic value of HRV in both symptomatic and asymptomatic individuals [38,39]. Thus, wearable devices capable of HRV analysis may aid in identifying CCS patients at higher risk for complications and disease progression.

Despite its advantages, motion artifacts remain a significant limitation of PPG. However, recent developments in advanced signal processing algorithms have significantly improved their robustness and reliability in ambulatory settings [40,41].

##### Phonocardiogram (PCG)-Based Sensors

PCG involves recording acoustic signals generated by the heart, typically through a sensor or microphone, and converting them into graphical representations. Although not originally intended for coronary assessment, PCG has garnered increasing interest as a non-invasive modality capable of detecting abnormal heart sounds generated by turbulent blood flow through partially occluded coronary arteries.

When paired with machine learning (ML) algorithms, PCG has demonstrated promising results in CAD screening. For example, a wearable vest incorporating multichannel PCG sensors achieved a diagnostic accuracy of approximately 80% in under two minutes during CAD screening [42]. Furthermore, advanced AI models based on multimodal data integration, combining ECG, PCG, and derived coupling signals, have shown improved accuracy in differentiating patients with and without CCS [43].

#### 2.2.2. Implantable Sensors

Implantable devices provide high-resolution, continuous monitoring and are particularly valuable in patients with known CCS or those at high risk of disease progression.

##### Insertable Cardiac Monitors (ICMs)

ICMs are subcutaneous devices capable of long-term, single-lead ECG monitoring. Their advantages include minimal motion artifacts and battery life lasting up to three years. While primarily validated for arrhythmia detection (as demonstrated in the CRYSTAL AF and STrOKE-AF studies), ICMs also detect ischemia-induced arrhythmias and increased ventricular ectopy, offering indirect assessment of myocardial ischemia [44,45,46].

##### Intracardiac Devices (ICDs and CRT-Ds)

Modern ICDs and cardiac resynchronization therapy defibrillators (CRT-Ds) now incorporate diagnostic features such as continuous ST-segment monitoring, enabling early detection of silent ischemia with greater sensitivity than surface ECGs [47]. The AngelMed Guardian^®^ System (Angel MedicalSystems, Eatontown, New Jersey, NJ, USA) is the first FDA-approved implantable device specifically designed for real-time ischemia detection via intracardiac electrogram analysis every 90 s. The ALERTS trial showed a median time to hospital presentation of 51 min with Guardian use, compared to over 30 h in control groups relying solely on symptom recognition [48]. Subsequent case reports further supported the device’s utility in detecting atypical ischemia, such as myocardial bridging [49].

##### Impedance Monitoring

ICDs and CRT-Ds also enable intrathoracic impedance monitoring to detect early signs of fluid overload, indicative of impending HF, which often coexists with CCS. The Medtronic OptiVol™ (Medtronic Inc., Minneapolis, MN, USA) system combines impedance data with clinical markers to anticipate decompensation. While the FAST trial reported a sensitivity of 76% for predicting HF events, the OptiLink HF study did not demonstrate a significant reduction in hospitalizations or mortality [50,51].

#### 2.2.3. Smart Stents

Smart stents increasingly incorporate pressure sensors within stent scaffolds to monitor in-stent restenosis in real time. These systems wirelessly transmit telemetry data, enabling early detection of reocclusion [52]. Some devices, tested in porcine models, utilize changes in device resonance and frequency response to local blood pressure variations [53]. Other smart stents provide data on restenosis risk based on flow velocity and have been tested in thoracic aorta models [54]. One model evaluated in rats demonstrated the capacity to detect in-stent restenosis while also providing insights into cardiac functional dynamics [55].

#### 2.2.4. Biochemical Sensors

Biochemical sensors are emerging as powerful tools for the early detection and ongoing management of ischemia in CCS patients. They continuously monitor biomarkers of myocardial stress and injury, such as cardiac troponins, ischemia-modified albumin (IMA), lactate, and reactive oxygen species (ROS).

These sensors typically employ electrochemical or optical detection and can identify ischemia even prior to observable ECG changes [56]. A flexible wearable troponin I sensor developed by Sengupta et al. demonstrated excellent sensitivity for continuous monitoring [57]. Additionally, a gold nanoparticle-based point-of-care IMA sensor showed rapid ischemia detection [58]. Multiplexed lab-on-chip systems now allow simultaneous measurement of lipid profiles and troponin levels from a single blood sample, improving both diagnosis and risk stratification [59].

#### 2.2.5. Exhaled Breath Analysis

Exhaled breath analysis using gas chromatography–mass spectrometry (GC-MS) identifies volatile organic compounds (VOCs) that reflect metabolic changes related to myocardial ischemia. VOC profiles may serve as non-invasive biomarkers of CCS. In a proof-of-concept study, breath analysis achieved 100% sensitivity in detecting significant coronary lesions requiring intervention [60]. Another study utilized a portable electronic nose (eNose) system to analyze exhaled breath in patients with acute chest pain, successfully differentiating between obstructive and non-obstructive coronary artery disease [61]. The BIONOTE-V device (Campus Bio-Medico University, Rome, Italy) demonstrated a sensitivity of 78.3% and specificity of 68.4% in distinguishing CAD severity, with VOC profiles correlating to SYNTAX scores and suggesting potential use in anatomical risk stratification [62].

**Table 1:** Type of sensors, technology used, features, clinical applications, and key studies. AF (Atrial Fibrillation), AI (artificial intelligence), CAD (Coronary Artery Disease), CCS (Chronic Coronary Syndrome), CRT-D (Cardiac Resynchronization Therapy Defibrillator), ECG (electrocardiogram), HR (heart rate), HRV (heart rate variability), ICD (Implantable Cardioverter Defibrillator), ICM (Insertable Cardiac Monitor), IMA (ischemia-modified albumin), ML (machine learning), PCG (phonocardiography), PPG (photoplethysmography), ROS (reactive oxygen species), STEMI (ST-Elevation Myocardial Infarction), VOCs (volatile organic compounds), and GC-MS (gas chromatography–mass spectrometry).

Sensor Type	Technology Used	Features	Clinical Applications	Key Studies
**Wearable Sensors**	ECG, PPG, PCG	Non-invasive, continuous monitoring; embedded in smartwatches, patches, etc.	Arrhythmia, ischemia, heart rate variability	
ECG-based	Electrodes measuring the heart’s electrical signals	Detects ST-segment changes, arrhythmias, AF detection	Arrhythmias, myocardial infarction detection	Barrett et al. (2014) [33], Spaccarotella et al. (2020) [34], Jung et al. (2024) [35], Turakhia et al. (2019) [21]
PPG-based	LED + photodetector, measures blood volume changes	HR and HRV monitoring; limited by motion artifacts	Long-term autonomic profiling, CAD prognosis	Kotecha et al. (2012) [38], Miller et al. (2022) [39], Vicente-Samper et al. (2023) [40]
PCG-based	Microphones detecting heart sounds	Detects murmurs, turbulent flow; enhanced by ML/AI	Coronary artery screening, differentiation of CCS vs. non-CCS	Fynn et al. (2025) [42], Sun et al. (2024) [43]
**Implantable Sensors**	ICMs, ICDs, CRT-Ds, impedance monitoring	Long-term, high-resolution ECG and impedance monitoring	Arrhythmia detection, ischemia, heart failure prediction	
Insertable Cardiac Monitors (ICMs)	Subcutaneous ECG monitors	Single-lead, multi-year monitoring	Arrhythmia detection, indirect ischemia monitoring	Sanna et al. (2014) [44], Bernstein et al. (2021) [45], Giancaterino et al. (2018) [46]
ICDs / CRT-Ds	Implanted defibrillators with ST-segment + impedance monitoring	Real-time ischemia detection; fluid overload monitoring	Silent ischemia, heart failure	Fischell et al. (2010) [47], Gibson et al. (2019) [48], Rao et al. (2024) [49]
Impedance monitoring	Intrathoracic impedance sensing (in ICDs/CRT-Ds)	Detects fluid overload; combined with clinical data for heart failure prediction	Heart failure risk and early decompensation	Abraham et al. (2011) [50], Böhm et al. (2016) [51],
**Smart Stents**	Pressure/flow sensors embedded in stents	Wireless telemetry, resonance detection	In-stent restenosis detection, flow dynamics	X. Chen et al. (2014) [53], Kim et al. (2022) [52], Chaparro-Rico et al. (2020) [54], Oyunbaatar et al. (2023) [55]
**Biochemical Sensors**	Electrochemical/optical sensors	Detect troponin, IMA, lactate, ROS; lab-on-chip integration	Early ischemia detection, risk stratification	Sengupta et al. (2023) [57], Li et al. (2013) [58], Wu et al. (2017) [59], Q. Chen et al. (2023) [56]
**Exhaled Breath Analysis**	GC-MS, eNose, VOCs profiling	Non-invasive, detects VOCs related to ischemia	CCS detection, obstructive vs. non-obstructive CAD, risk stratification	Lombardi et al. (2024) [60], Nardi Agmon et al. (2022) [61], Segreti et al. (2020) [62]

### 2.3. Routinary Use in Clinical Practice

While the utility of wearable sensor technologies in monitoring cardiovascular risk and supporting rehabilitation in CCS patients is well established, their incorporation into standardized diagnostic protocols remains an evolving process. Emerging evidence suggests that biosensor-equipped devices can identify CCS-related abnormalities by continuously tracking physiological variables, yet large-scale validation confirming their diagnostic and prognostic efficacy remains limited.

Currently, wearable and biosensor technologies are primarily utilized for lifestyle monitoring, secondary prevention optimization, and rehabilitation support in patients with known cardiovascular disease. These tools also offer potential in guiding personalized follow-up, especially in cases of stable or non-obstructive coronary disease.

Robust evidence supports the role of these technologies in cardiovascular risk factor modification. A meta-analysis by Widmer et al. [63], encompassing over 51 clinical trials, showed that digital health interventions, many incorporating sensor-based monitoring, led to significant improvements in blood pressure, cholesterol, body mass index (BMI), and physical activity in at-risk and affected individuals. A more recent umbrella review in The Lancet Digital Health [64] involving over 160,000 participants confirmed that wearable activity trackers increased daily step counts and reduced adiposity and systolic blood pressure, supporting their role in CCS secondary prevention.

Beyond lifestyle changes, wearable devices enhance medication adherence and enable longitudinal self-monitoring. A meta-analysis in JAMA Network Open [65] found that wearable activity trackers, when combined with clinician support, significantly improved physical activity levels and treatment adherence in patients with cardiometabolic risk. These platforms often include user-friendly dashboards that foster patient engagement and behavioral change, central elements in chronic disease management.

Their clinical potential has also been supported by interventional trials. The CHANGE study [66], a multicenter randomized trial, evaluated a smartphone-guided secondary prevention program in CCS patients. After 12 weeks, participants in the intervention group experienced a mean systolic blood pressure reduction of 15.5 mmHg, compared to 6.0 mmHg in the standard care group, highlighting the efficacy of digitally supported sensor-based strategies.

Wearable technologies are also redefining follow-up strategies for CCS patients. Continuous data acquisition may serve as a digital biomarker of disease stability or decompensation, allowing for individualized therapeutic adjustments, particularly in patients with intermittent or subclinical angina.

In cardiac rehabilitation (CR), digital solutions are addressing historical participation barriers. The REMOTE-CR trial demonstrated that sensor-assisted telerehabilitation programs can match center-based models in improving functional capacity, medication adherence, and quality of life post-myocardial infarction or revascularization [67]. In another study, the VITALITY-HFpEF trial was conducted using a wearable accelerometer that measured continuous physical activity in patients with HFpEF and showed that this was well below the age-adjusted values for normal adults. However, this trial demonstrated no statistically significant association between physical activity and intensity derived from accelerometers and Kansas City Cardiomyopathy Questionnaire-Physical Limitation Score (KCCQ-PLS) or 6 min walk test (6-MWT) [68].

While the 2024 ESC Guidelines emphasize mobile health (mHealth) primarily for promoting behavioral changes and adherence, they also advocate further research into the diagnostic and prognostic utility of remote technologies in CCS [1]. Future integration into clinical decision-making pathways will depend on robust validation and regulatory consensus.

Ongoing trials are currently assessing the feasibility, safety, and clinical utility of biosensor technologies in real-world CCS settings. These studies aim to identify optimal integration points across the care continuum, from initial triage to risk stratification and long-term monitoring [69].

## 3. Controlling Risk Factors and Monitoring for the Detection of Disease Progression

Once a diagnosis of CCS is made, strict follow-up for risk factor control, adherence to medical therapy, and surveillance for signs and symptoms of disease progression becomes central to patient management. However, adherence to long-term therapy in chronic conditions remains a major issue. Evidence shows that in high-income countries, adherence averages only around 50%, and is even lower in developing regions [70]. Furthermore, data from the ESC-EORP EUROASPIRE V registry reveal that many CCS patients continue to engage in unhealthy behaviors such as smoking, poor dietary habits, and physical inactivity [71]. Therefore, interventions aimed at promoting lifestyle changes and improving cardiometabolic health in high-risk populations, such as CCS patients, are a critical priority for clinicians.

The most recent CCS guidelines strongly endorse a multidisciplinary approach to enhance adherence to medical therapy and improve patient outcomes. The use of mHealth and eHealth tools is recommended and may play an important role in this context [1]. These interventions have proven effective in encouraging healthy behaviors but are less successful in reducing unhealthy behaviors, such as smoking, alcohol use, sedentary lifestyle, and poor diet, and in improving clinical outcomes [72].

Commercially available wearable devices designed to encourage physical activity offer a practical and accessible solution for promoting lifestyle changes. Their widespread use makes them ideal tools for large-scale interventions. A review of 35 randomized controlled trials involving 8147 participants with various chronic conditions, including type 2 diabetes, cardiovascular diseases, and obesity, demonstrated that consumer-grade activity trackers led to reductions in waist circumference, systolic blood pressure, and LDL cholesterol [73]. Another study found that incorporating activity trackers into secondary prevention programs for CCS patients improved physical fitness, reduced the risk of major adverse cardiovascular events (MACE), and enhanced quality of life. However, the effect on LDL-C levels remained inconclusive [74].

Among CCS risk factors, diabetes mellitus is one of the most significant contributors to CAD onset and progression, especially when glycemic control is suboptimal. The OPTIMAL randomized clinical trial investigated the effect of CGM-guided glycemic control on coronary atherosclerosis progression in type 2 diabetic patients with CAD. However, the study found no significant difference compared to standard HbA1c-guided control [75].

Surveillance for disease progression in CCS is essential during follow-up and is typically performed through risk stratification and the identification of symptoms suggestive of CAD progression. However, in patients with stable angina or occult myocardial ischemia, conventional symptom-driven evaluations may fail to detect clinically relevant events. For example, coronary stent restenosis is a common and often silent complication of angioplasty. As previously noted, certain “smart” stents have been developed to detect restenosis through various mechanisms, often involving pressure sensors. Other devices, such as soft implantable bioelectronic systems capable of wireless continuous monitoring, have also been tested in vitro [76].

## 4. Assessing Treatment Response

### 4.1. Real-Time Feedback on the Efficacy of Pharmacological or Interventional Treatments

Biosensors, both wearable and implantable, now enable dynamic, longitudinal monitoring of cardiac function, allowing for timely therapeutic adjustments based on physiological and biochemical feedback.

Wearable electrocardiographic biosensors, such as adhesive smart patches and sensor-integrated textiles, provide continuous ambulatory monitoring of cardiac electrophysiology. These systems can detect subclinical ischemic episodes, arrhythmias (e.g., AF, premature ventricular contractions), and changes in heart rate variability (HRV), a parameter sensitive to autonomic tone and myocardial ischemia. Clinical trials have shown that continuous ECG monitoring via wearable devices enhances early detection of transient ischemic events and silent arrhythmias, enabling clinicians to more effectively evaluate and adjust therapies such as beta-blockers, calcium channel blockers, and rate-control agents in CCS patients [27,77].

Implantable hemodynamic sensors, such as the CardioMEMS HF System (St. Jude Medical, Inc., Atlanta, GA, USA), provide telemetric measurement of pulmonary artery pressure (PAP), a surrogate for left-sided filling pressures and cardiac preload. This technology has been validated in randomized trials (e.g., CHAMPION) for reducing hospitalizations in HF patients through early titration of diuretics and vasoactive medications [50,78]. Given the frequent overlap between CCS and HF phenotypes, particularly in patients with reduced ejection fraction or post-infarction remodeling, integrating PAP data into therapeutic algorithms facilitates personalized management and may prevent clinical deterioration.

Beyond electrophysiologic and hemodynamic parameters, biochemical biosensors represent valuable tools for assessing pharmacologic response and disease activity at the molecular level. Microneedle platforms and epidermal biosensors capable of sampling interstitial fluid or sweat provide near-continuous, minimally invasive measurement of key biomarkers such as high-sensitivity cardiac troponins (hs-cTnI/T), NT-proBNP, lactate, and inflammatory cytokines (e.g., IL-6, TNF-α) [79,80]. These biomarkers can guide decisions on antiplatelet therapy, lipid-lowering agents (e.g., statins, PCSK9 inhibitors), and anti-inflammatory strategies.

Furthermore, integrated biosensor-enabled stents and vascular scaffolds represent a promising frontier in post-PCI monitoring. These devices are embedded with microelectromechanical systems (MEMSs) capable of measuring local flow dynamics, endothelial shear stress, and signs of in-stent restenosis or re-endothelialization. Early data suggest these tools may enable prompt detection of mechanical complications and support timely pharmacologic or procedural interventions [81,82].

### 4.2. Personalized Therapy Based on Biosensor Readings

The incorporation of biosensor-generated data into clinical workflows is ushering in a shift from population-based, reactive management to precision-guided, proactive cardiovascular care. In CCS, continuous physiological and biochemical monitoring allows for real-time therapy adjustments tailored to individual patient profiles, including variations in disease phenotype, drug responsiveness, and comorbidities.

Large-scale, high-frequency data from wearable and implantable sensors are increasingly analyzed using AI and ML algorithms. These tools can detect patterns and anomalies not evident to clinicians, offering predictive insights into treatment efficacy and potential clinical deterioration. For example, AI-enhanced ECG interpretation has been shown to identify AF signatures even from sinus rhythm tracings, enabling earlier risk stratification and intervention [83].

Home-based hemodynamic monitoring platforms also support personalized medication management, especially in patients with overlapping hypertensive heart disease, autonomic dysfunction, or frailty. Titrating beta-blockers, vasodilators, and diuretics based on hemodynamic indices can improve symptom control and reduce ischemic burden [84]. Though widely adopted in HF and AF, this strategy is increasingly applicable to aging, multimorbid CCS populations.

Risk factor control remains essential in CCS. A study by Treskes et al. evaluated the feasibility and effectiveness of smart technologies for blood pressure control in 200 post-MI patients. Participants used smartphone-compatible devices to record daily single-lead ECGs, blood pressure, weight, and step count. While feasible and well accepted, the intervention did not significantly improve blood pressure control compared to usual care [85].

An added dimension of personalization can be achieved through integration with pharmacogenomics. For instance, CYP2C19 loss-of-function alleles impair clopidogrel activation, increasing thrombotic risk after PCI. Real-time biosensor-based assessment of platelet reactivity or thrombo-inflammatory biomarkers could inform early switching to agents like ticagrelor or prasugrel in poor responders [86]. This pharmacodynamic feedback brings clinical care closer to genotype–phenotype concordance.

The increasing use of mHealth platforms that consolidate biosensor outputs into cloud-based dashboards and mobile apps further democratizes access to health data. These systems allow patients to track trends in activity, medication adherence, and vital signs, fostering engagement and shared decision-making. Randomized studies have shown that mHealth-enhanced care improves adherence, reduces emergency visits, and improves outcomes in cardiovascular populations, including AF and HF [32].

Table 2 summarizes the clinical utility, sensor types involved, and current limitations across the various management stages of CCS.

## 5. Future Perspectives and Innovations

### 5.1. Nanotechnology, Advanced Materials, and Miniaturization

The integration of nanotechnology, advanced materials, and miniaturization has significantly advanced sensor technologies. Most of the sensors currently used in healthcare are miniaturized to enhance patient adherence, minimize interference with daily activities, and reduce aesthetic impact, without compromising their functional capabilities. Their potential has also been explored in the context of CCS [87].

Various types of wearable sensors have been developed, such as wearable patches [6,88,89], epidermal tattoos [90,91], and even “electronic noses” [61], even when not based on nanotechnology and not wearable, capable of analyzing volatile organic compounds in a non-structured environment [62], among others [92]. Nanotechnology refers to the manipulation and application of materials at the nanoscale, typically between 1 and 100 nanometers. At this level, materials exhibit unique physical, chemical, and biological properties that differ significantly from their bulk counterparts, including enhanced surface area, reactivity, electrical conductivity, and mechanical strength, making them ideally suited for sensitive and selective detection in sensors.

Nanomaterials now allow for lower detection limits than previously possible, enabling even single-molecule detection [93]. Graphene and carbon nanotubes, due to their excellent electrical conductivity, are used in electrochemical biosensors to enhance electron transfer, allowing for faster and more precise detection of analytes in complex biological samples such as blood and saliva. These platforms enable quantitative detection of cancer-related biomarkers, such as DNA, miRNA, small molecules, and proteins [94]. The development of nanoengineered multichannel immunosensors employing unsupervised clustering algorithms represents a promising leap forward in acute thrombotic event detection. As demonstrated by Wang et al., these fiber-laser–engraved carbon-nanotube sensors simultaneously quantify multiple circulating biomarkers and can predict acute blood clot formation and thrombotic events, a promising technology for early detection of plaque instability and microthrombosis [95].

Particularly promising is the use of aptamers, short nucleic acid sequences with high affinity and specificity toward targets like troponin I, combined with electrochemical immunoassay technologies. These sensors, which are highly stable and minimally immunogenic, may offer an efficient alternative to monoclonal antibodies. Highly sensitive aptasensors for TnI could thus be applied for the early and accurate diagnosis of myocardial ischemia [96].

Furthermore, real-time wearable nanosensors could be integrated with drug delivery systems, allowing for on-demand release of therapeutics [97]. In therapy, nanoparticles can enable targeted drug delivery to pathological areas; for instance, statin-loaded nanoparticles directed to atherosclerotic plaques, reducing systemic side effects and enhancing efficacy [98]. Other approaches use enzyme-mimetic nanoparticles to eliminate ROS [99], or nanoparticles to deliver natural antioxidants with poor bioavailability (e.g., curcumin, resveratrol), offering promising strategies to modulate oxidative stress and inflammation, key contributors to atherosclerosis and adverse cardiovascular outcomes [100].

Among the most commonly used nanomaterials are gold nanoparticles (AuNPs), known for their excellent biocompatibility and optical properties. They are often applied in colorimetric sensors and are widely investigated for biomarker detection, especially in cancer surveillance [101].

### 5.2. Integration with Artificial Intelligence and Machine Learning

The integration of sensors, sensor data, and AI has the potential to revolutionize healthcare, especially in the management of chronic diseases. Both ML and deep learning (DL) are branches of AI; they are based on complex mathematical models and neural networks that can mimic the intelligence of human learning and problem-solving for complex tasks. The volume, velocity, and variety of data render traditional analytic methods inadequate, hence the growing role of AI in processing, interpreting, and deriving actionable insights from complex, high-dimensional datasets. Recent advancements in machine learning-integrated point-of-care sensors have significantly enhanced the early detection and continuous monitoring capabilities for cardiovascular conditions, offering promising applications in their management [102]. The application of DL to CCTA has allowed more accurate quantification of atherosclerotic plaques and stenoses, as well as better long-term cardiovascular risk stratification, as shown in a multicenter study by Lin et al. The results suggest that AI-based approaches may reduce the need for invasive procedures and enhance early diagnosis, facilitating timely and personalized therapeutic decisions [103]. Wearable sensors, such as continuous glucose monitors, ECG patches, and smart inhalers, collect real-time physiological data, enabling continuous monitoring of patients’ health status. These sensors provide a wealth of data that, when processed and analyzed, can offer insights into disease progression, treatment efficacy, and patient behavior. AI and ML algorithms are employed to analyze this vast amount of data, identifying patterns and making predictions that assist healthcare providers in making informed decisions. The application of a DL approach to ECG interpretation, for example, resulted in the diagnosis of a broad range of distinct arrhythmias from single-lead ECGs with high diagnostic performance similar to that of cardiologists and has the potential to reduce the rate of misdiagnosed computerized ECG interpretations and improve the efficiency of expert human ECG interpretation [104]. In chronic disease management, such as diabetes, cardiovascular diseases, and chronic obstructive pulmonary disease (COPD), the integration of these technologies facilitates personalized care. For instance, AI-powered mobile applications are capable of providing personalized feedback and coaching to help patients track their medication, sleep, exercise, and other health behaviors [105]. The use of AI extends to wearable devices that monitor vital signs such as oxygen levels, respiratory rates, and heart rhythms. These devices, equipped with sensors and AI algorithms, can detect anomalies and alert patients and healthcare providers to potential issues, enabling timely interventions. For example, smart inhalers equipped with sensors track medication use and provide feedback to patients, improving asthma management [106]. AI also plays a crucial role in predictive analytics. By analyzing historical and real-time data, these technologies can predict disease exacerbations, hospital readmissions, and other critical events, allowing for proactive management and reducing healthcare costs. ML models, such as random forests and support vector machines, have been utilized to predict hypoglycemia in diabetic patients based on continuous glucose monitoring data, demonstrating high accuracy and reliability [105].

AI and ML algorithms could also be employed in analyzing the vast amounts of data generated by wearable sensors to identify patterns indicative of cardiovascular issues. The integration of sensor-derived data with AI and ML algorithms offers the potential for early detection, continuous monitoring, and personalized therapy. For instance, AI models have been developed to detect HF with reduced ejection fraction (HFrEF) using two-lead ECG data from smartwatches. A study demonstrated that an AI algorithm could accurately identify HFrEF with reasonable performance, using data from over 137,000 patients. This capability allows for early diagnosis and intervention, potentially preventing severe complications [107]. Recent studies have demonstrated the efficacy of artificial neural networks in analyzing ECGs and echocardiograms for the automatic detection of abnormalities [108]. Algorithms based on ML demonstrated the potential to be applied in the diagnosis of CCS and MI, proposing decision-making systems that could help physicians in daily clinical practice [109,110,111].

AI and neural network algorithms can also be useful in predicting 1-year mortality and admission to hospital for HF after an acute myocardial infarction, allowing the identification of patients at risk for future cardiovascular events and giving the possibility to tailor therapies and follow-up [112]. The VOCs analysis with machine-learning models demonstrated to discriminate between patients with vs. without CCS [113].

Despite the advancements, challenges remain in the integration of sensors and AI in healthcare. Issues such as data privacy, security, and interoperability need to be addressed to ensure the effective and ethical use of these technologies. Moreover, the accuracy of sensor data can be influenced by factors like sensor calibration, placement, and environmental conditions, which can impact the reliability of AI and ML predictions. At present, no registered clinical trials appear to specifically investigate the integration of artificial intelligence into the clinical management of CCS.

### 5.3. Remote Control and Telemedicine

The use of sensors and their application in telemedicine for remote control of patients suffering from chronic conditions has the potential to reshape traditional models of care by enabling continuous, remote patient monitoring and facilitating the integration of telemedicine into clinical practice. Sensors, when linked to telemedicine platforms, transmit data to cloud-based systems where they can be accessed by healthcare providers for timely review and intervention [114]. The most recent European Guidelines for the management of Chronic Coronary Syndromes recommend the use of mobile health interventions (e.g., SMS, apps, and wearable devices) to enhance adherence to therapy and promote healthy lifestyles (Class I recommendation, Level A evidence) [1].

In the case of AF diagnosis, for example, immediate monitoring with a home-based wearable ECG sensor patch, compared with delayed monitoring, showed the potential to improve AF diagnosis after 4 months; this finding could reduce the complications of an undiagnosed AF [27]. In HF management, several sensors have been validated in a telemedicine setting; they can help cardiologists in promoting patient adherence, individualize guideline-directed medical therapies, and identify fluid overload status or a worsening of the cardiac function [115]. In rural or underserved areas, where access to specialist cardiology care is limited, telemedicine-supported remote monitoring using sensors can bridge the healthcare gap, offering continuous supervision. For example, an interesting study has demonstrated that a multichannel smartwatch ECG (recording leads I, II, III, V1, V2, V3, V4, V5, and V6) could be capable of detecting ECG alterations and their localization similar to those noted with a standard 12-lead ECG in patients with acute coronary syndromes. This finding may have the potential for earlier diagnosis of ACS and a prompt intervention by emergency medical services and a rapid dispatch to the correct clinical setting in patients at risk for acute coronary events, for example, those suffering from CCS [34].

**Figure 1:**
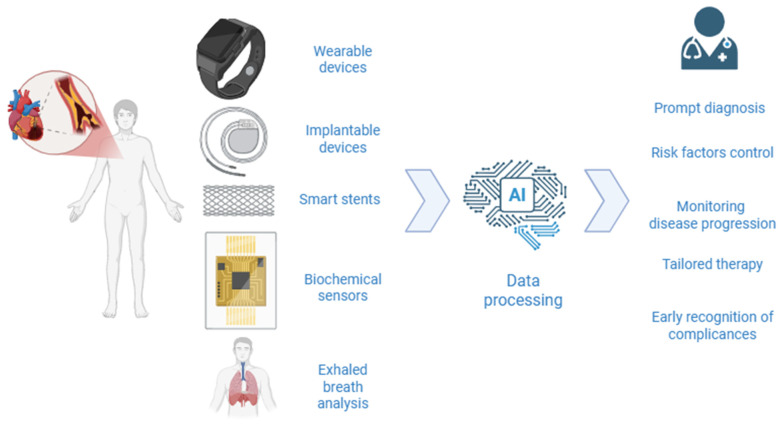
Example of utilizing sensors for the management of CCS. Sensors generate a large amount of data that are processed by artificial intelligence and interpreted by a physician.

The combination of sensors and AI holds the potential to decentralize the management of CCS, moving much of the monitoring and decision-making from hospital settings to primary care and home environments. By leveraging cloud-connected devices and remote data analysis, healthcare providers can track patient status in real time, intervene promptly when risk thresholds are crossed, and reduce the need for hospital-based diagnostics. This territorial approach not only makes care more accessible and less burdensome for patients but also alleviates pressure on specialized cardiology centers, fostering a more sustainable and scalable model of chronic disease management. Figure 1 represents a schematic example of how sensor data can be collected and processed by AI to facilitate and enhance CCS management.

Despite promising advances, there remains a significant lack of dedicated sensor devices specifically designed for their integration in telemedicine for the management of CCS, as well as a shortage of large-scale randomized clinical trials validating their clinical efficacy and impact on patient outcomes. The TELSINCORC trial is an ongoing multicenter, randomized study evaluating whether a telemonitoring system integrated with a mobile app can improve functional capacity and continuity of care in patients with Chronic Coronary Syndrome [116].

## 6. Current Challenges and Limitations

### 6.1. Potential for False Positives/Negatives in Biosensor Readings and Consequences in Clinical Practice

A major challenge in the clinical application of sensor technologies for the early detection and diagnosis of CCS lies in the variable accuracy and reliability of the data they generate. This issue is particularly prominent in wearable devices, which currently represent the most widely adopted form of biosensor technology.

Compact ECG-based sensors seem to show high diagnostic accuracy in detecting ischemic changes. In the 3-lead ECG system by Shvilkin et al. (2023), comparative analysis (using a baseline ECG) achieved sensitivity and specificity both over 90% in detecting balloon-induced coronary occlusion, nearly equivalent to full 12-lead ECG performance, whereas spot (single-point) readings dropped significantly, underlining the value of baseline comparison [23]. Similarly, in the study by Spaccarotella et al. (2020), a multichannel smartwatch ECG system matched standard 12-lead ECG in detecting ST-segment elevation (sensitivity 93%, specificity 95%) and non-ST elevation abnormalities (sensitivity 94%, specificity 92%) [34].

However, false-positive readings may result from both hardware- and user-related factors. In wearable ECG devices, issues such as motion artifacts, variable skin impedance, and suboptimal electrode contact can distort signal acquisition, mimicking arrhythmias or ST-segment deviations. For instance, the Apple Heart Study reported that approximately 20% of participants who received irregular rhythm notifications had no clinically relevant arrhythmias upon subsequent ECG patch evaluation [32].

PPG-based systems, commonly found in smartwatches, face similar limitations. These sensors, which estimate heart rate by detecting light absorption fluctuations linked to blood volume changes, are particularly susceptible to signal noise introduced by physical activity, skin perfusion variability, or improper device placement [117]. Moreover, systematic bias in PPG interpretation has been observed in individuals with darker skin tones, where increased melanin content interferes with optical signal transmission, potentially compromising accuracy [118,119]. While some studies question the clinical relevance of this bias [120], the risk of skewed diagnostics across diverse populations remains non-negligible.

False-positive outputs may result in unnecessary emergency department visits, biomarker testing, and even invasive procedures such as coronary angiography, outcomes that are particularly burdensome in asymptomatic or low-risk individuals. These events impose significant financial, psychological, and procedural burdens, especially when device alerts lack an appropriate clinical context.

Conversely, false-negative readings pose a more insidious threat. Undetected ischemic episodes or arrhythmias due to signal dropout, limited lead configurations, or insufficient algorithm sensitivity may lead to false reassurance and delayed intervention. This is especially dangerous in CCS, where silent myocardial ischemia and paroxysmal AF are frequently asymptomatic but clinically significant [121]. Furthermore, wrist-worn heart rate monitors have demonstrated reduced accuracy in individuals with higher BMI, darker skin tones, and in male patients. The type of physical activity also influences performance, with greater accuracy during cycling and poorer reliability during walking [122].

These diagnostic shortcomings arise not only from hardware limitations but also from algorithmic deficiencies. Many biosensor platforms rely on proprietary ML models trained on narrowly representative datasets, which often lack adequate clinical diversity. The overrepresentation of young, healthy, and predominantly white populations in training datasets significantly undermines performance in older adults, patients with comorbidities, and ethnically diverse groups [123,124].

Another complication stems from the opacity of diagnostic algorithms. Most consumer-grade sensors do not disclose the criteria or thresholds used to generate alerts, nor do they report false-alarm rates. This lack of transparency erodes clinical trust and presents a critical barrier, particularly in resource-limited or primary care settings, where specialist oversight is minimal or absent.

A further limitation is the lack of contextual integration. Many current biosensor systems operate independently of the broader clinical picture, increasing the risk of misclassification. For example, transient ST-segment changes during exertion may be physiological rather than ischemic, yet may be misinterpreted without correlation to symptoms, biomarkers, or imaging findings [19].

Finally, the medico-legal implications of diagnostic inaccuracies remain underexplored. As sensor-based technologies become increasingly integrated into clinical workflows, questions of liability, whether due to missed diagnoses (false negatives) or unwarranted interventions (false positives), must be addressed.

In summary, while biosensor technologies hold great promise for improving cardiovascular care in CCS, their current limitations in signal fidelity, algorithm robustness, and clinical contextualization must be resolved. Addressing these gaps will require continued technical innovation, such as advanced noise-reduction algorithms and multimodal data integration, supported by rigorous clinical validation across diverse populations. Moreover, transparent algorithm development, robust regulatory oversight, and standardized data interoperability protocols will be essential to ensure both the safety and efficacy of future sensor-based diagnostics in cardiology.

### 6.2. Economic Considerations for Widespread Adoption and Availability in Low-Resource Settings

The large-scale implementation of sensor technologies in healthcare presents significant economic and structural challenges, particularly in low- and middle-income countries (LMICs). Equitable access across heterogeneous healthcare systems remains a major concern due to disparities in infrastructure, resource availability, and technological preparedness.

The cost of biosensor technologies extends well beyond hardware acquisition. Implantable monitors, for instance, require surgical procedures, specialized monitoring equipment, and secure data transmission platforms. Even relatively accessible devices, such as ECG patches or consumer-grade smartwatches, depend on robust backend infrastructure for data storage, processing, and integration into electronic health records (EHRs). These requirements necessitate substantial investments in digital infrastructure, cybersecurity, cloud computing, and specialized training, which may be prohibitively expensive for underfunded health systems [125,126].

From a health equity perspective, there is growing concern that sensor-based cardiology may widen, rather than bridge, existing disparities. In both LMICs and underserved populations within high-income countries, limited access to smartphones, internet connectivity, and digital health literacy may prevent entire patient cohorts from benefiting from remote monitoring technologies. These barriers highlight the importance of ensuring accessibility, affordability, and user-friendliness of digital health tools across all care settings [127]. Moreover, sensor-based technologies are often concentrated in urban tertiary centers, leaving primary care facilities, where early CCS detection is most impactful, undersupplied or excluded.

Sustainability represents another key consideration. Wearable medical-grade sensors require regular maintenance, including recalibration, battery replacement, and software updates. Implantable devices, such as insertable cardiac monitors (ICMs) and smart stents, generally have a battery lifespan of 2–4 years and require specialized expertise for both implantation and replacement. In combination with the rapid pace of technological obsolescence, these factors present logistical and financial barriers for health systems pursuing long-term, scalable monitoring solutions.

Environmental sustainability must also be addressed. A recent review highlighted the significant electronic waste produced by short-lifecycle wearable devices, citing poor repairability and a lack of standardized recycling programs as major concerns [128]. When deployed at scale without provisions for reuse or modular upgrading, these devices may exacerbate the environmental impact of digital health technologies.

Finally, the cost-effectiveness of sensor deployment for CCS screening and monitoring remains inadequately demonstrated. Although economic evaluations have shown favorable outcomes for sensor-based interventions in arrhythmia detection and management, particularly in AF, evidence supporting their utility in asymptomatic or low-risk CCS populations is limited. In these groups, low event incidence and uncertain diagnostic yield may not justify the high economic investment required for widespread adoption.

### 6.3. Need for Robust Algorithms and Platforms to Handle Large Volumes of Data

The clinical utility of sensor technologies is intrinsically tied to the capacity to process and interpret the vast volumes of data they produce. This is especially critical as the field transitions from point-of-care devices toward continuous monitoring platforms. Wearable and implantable cardiovascular sensors can generate thousands of data points per patient per hour. Over extended periods, this translates into millions of data entries per individual, surpassing the storage and analytic capabilities of conventional clinical infrastructures.

Currently, most electronic health record (EHR) systems are not equipped to manage real-time, high-frequency data streams. Designed primarily for episodic care documentation, they often fail to efficiently integrate outputs from biosensors, resulting in fragmented data housed across proprietary platforms and external cloud systems [19]. This fragmentation impairs clinical interpretation and continuity of care.

From a data management standpoint, the need for secure, high-capacity repositories is paramount. These systems must ensure compliance with data protection regulations while maintaining accessibility for clinical review and ML analysis. More critically, the challenge lies in interpreting continuous, high-dimensional data. Sensor outputs are frequently noisy, redundant, and context-sensitive, complicating the distinction between clinically actionable signals and benign physiological variation. Addressing this requires real-time signal processing pipelines and intelligent systems capable of prioritizing relevant events without overwhelming clinicians with false alarms.

AI and ML have become essential in navigating these challenges. DL frameworks, including convolutional neural networks (CNNs) and recurrent neural networks (RNNs), have demonstrated potential in automating arrhythmia detection, predicting myocardial infarction, and identifying early signs of HF decompensation [124,129]. However, their clinical utility depends on extensive training using large, heterogeneous datasets to ensure model generalizability.

A critical limitation in current AI deployment is the presence of bias within training data. Many algorithms are developed using datasets from high-income settings and predominantly white, male populations, which undermines performance in more diverse cohorts, including women, elderly individuals, and underrepresented ethnic groups. This is particularly concerning in CCS, where clinical presentations and disease trajectories may differ by sex and age. Despite the promising applications of AI, telemedicine, and cloud-based monitoring in CCS management, several challenges remain. Regulatory frameworks often lag behind rapidly evolving AI technologies, particularly for adaptive algorithms used in diagnostics. Ethical concerns include algorithmic bias, limited transparency in decision-making (“black-box” models), and potential over-reliance on automated outputs. Data privacy and cybersecurity are also critical issues, as wearable sensors and cloud platforms handle large volumes of sensitive patient information. Ensuring interoperability with clinical systems and maintaining patient trust will be essential for the safe and effective integration of these technologies into routine CCS care [123,130].

Equally important is the usability of analytic platforms. Effective clinical integration requires that analyzed data be presented in a timely, interpretable format, seamlessly embedded within routine workflows. Presently, few systems meet this standard, contributing to issues such as alert fatigue, diagnostic inertia, and cognitive overload [131].

**Figure 2:**
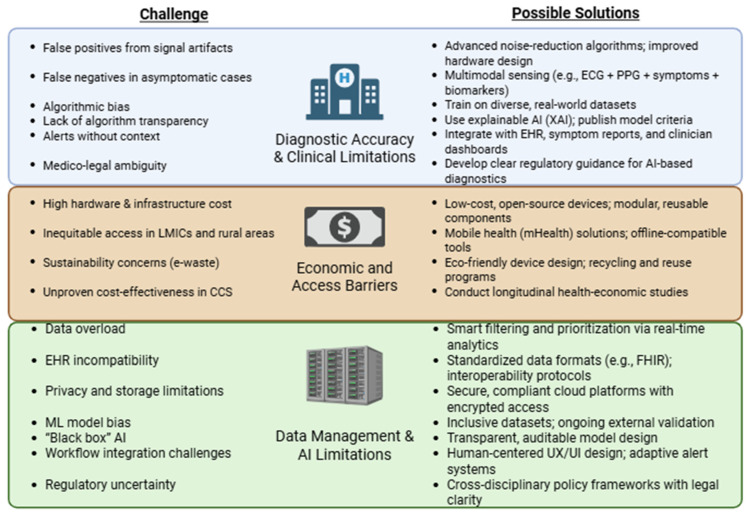
Barriers and Solutions in Sensor-Based CCS Monitoring: A Roadmap for Equitable and Effective Integration. CCS (Chronic Coronary Syndrome), ECG (electrocardiogram), PPG (photoplethysmography), BMI (body mass index), EHR (electronic health record), AI (artificial intelligence), ML (machine learning), XAI (explainable artificial intelligence), LMICs (low- and middle-income countries), mHealth (mobile health), CNN (convolutional neural network), RNN (recurrent neural network), FHIR (fast healthcare interoperability resources).

These technical and operational challenges are further compounded by legal and regulatory uncertainties. Real-time biometric analysis mandates stringent compliance with data privacy legislation, robust cybersecurity measures, and transparent consent protocols. Moreover, medico-legal accountability in cases of diagnostic error or delayed intervention due to AI-generated outputs remains poorly defined, raising concerns regarding liability and clinical governance [132]. Figure 2 summarizes the key challenges associated with sensor-based monitoring in CCS and outlines potential solutions to enhance their clinical utility, accessibility, and data integration.

## 7. Conclusions

Biosensors are playing an increasingly pivotal role in the diagnosis, monitoring, and management of various diseases, particularly in the field of cardiovascular medicine, where they offer a transformative approach to patient care. However, their application in the context of CCS remains relatively underexplored compared to other cardiovascular conditions, such as AF and HF.

By enabling real-time tracking of key physiological and biochemical parameters, wearable and implantable biosensors have the potential to facilitate earlier detection of ischemic events, provide more precise assessment of therapeutic responses, and support personalized treatment strategies. These technologies are contributing to a paradigm shift from reactive to proactive care, allowing continuous monitoring beyond traditional clinical settings and thereby improving patients’ quality of life. Their integration into routine clinical practice may also reduce the need for frequent hospital visits and invasive diagnostic procedures.

Despite these promising developments, several barriers continue to impede the widespread adoption of biosensor technologies in CCS management. Challenges include issues of measurement accuracy, long-term device reliability, biocompatibility, patient adherence, and the secure handling of large volumes of health data. Moreover, integration with existing healthcare infrastructure and the absence of standardized clinical protocols further complicate implementation. Regulatory uncertainties and ethical considerations around data privacy also require urgent attention.

Continued interdisciplinary research and development are essential to address these limitations and enhance the performance, safety, and accessibility of biosensors. Collaboration among clinicians, biomedical engineers, and data scientists will be key to tailoring sensor technologies for real-world clinical use. Finally, robust clinical validation through large-scale prospective trials will be critical to establish clinical efficacy, ensure safety, and build confidence among healthcare providers and patients.

## Figures and Tables

**Table 2:** Clinical utility of biosensors in the management stages of Chronic Coronary Syndrome (CCS). ECG (electrocardiogram), PPG (photoplethysmography), CGM (continuous glucose monitoring), BP (blood pressure), AI (artificial intelligence), mHealth (mobile health).

Management Phase	Clinical Objective	Types of Sensors Involved	Main Benefits	Current Limitations
Early Diagnosis and Risk Stratification	Detect silent ischemia, arrhythmias, and subclinical abnormalities	ECG patch, loop recorder, troponin sensors, PPG, consumer wearables	Early diagnosis, continuous monitoring, detection of silent ischemia, and dynamic risk stratification	Possible false positives/negatives, data heterogeneity, and low standardized clinical adoption
Disease Progression Monitoring	Assess therapy adherence, control risk factors, and detect signs of progression	Activity trackers, CGM, BP sensors, smart stents, multiparametric wearables	Increased patient engagement, early detection of restenosis, promotion of a healthy lifestyle, and continuous surveillance	Low effectiveness on harmful behaviors, response variability, and difficulty in data standardization
Therapeutic Response Evaluation	Monitor the effectiveness of pharmacological and interventional therapies	Wearable ECG, hemodynamic sensors, biochemical biosensors	Real-time feedback, therapy adjustment, detection of subclinical events, and prevention of hospitalizations	High costs, limited access in low-income settings, need for broader clinical validation
Personalized Therapies and Remote Management	Tailor treatments to individual characteristics and enable remote monitoring	AI + wearable/implantable sensors, mHealth, biosensors integrated with pharmacogenomics	Data-driven therapeutic decisions, patient involvement, home-based management, and reduced hospital burden	Lack of CCS-specific devices, need for randomized studies, privacy and security issues
Telemedicine and Territorial Accessibility	Overcome geographic barriers and ensure continuous follow-up	Multi-lead smartwatch, remote ECG, cloud platforms with patient dashboards	Reduced inequalities, monitoring in rural areas, early triage, improved care access, and efficient use of healthcare resources	Limited integration into clinical practice, lack of interoperable systems
